# Homozygous mutation in *DNAAF4* causes primary ciliary dyskinesia in a Chinese family

**DOI:** 10.3389/fgene.2022.1087818

**Published:** 2022-12-13

**Authors:** Guoliang Jiang, Lijun Zou, Lingzhi Long, Yijun He, Xin Lv, Yuanyuan Han, Tingting Yao, Yan Zhang, Mao Jiang, Zhangzhe Peng, Lijian Tao, Wei Xie, Jie Meng

**Affiliations:** ^1^ Department of Pulmonary and Critical Care Medicine, The Third Xiangya Hospital, Central South University, Changsha, China; ^2^ Hunan Key Laboratory of Organ Fibrosis, Central South University, Changsha, China; ^3^ Department of Nephrology, Xiangya Hospital, Central South University, Changsha, China; ^4^ Department of Cardiovascular Medicine, Xiangya Hospital, Central South University, Changsha, China

**Keywords:** primary ciliary dyskinesia, female infertility, DNAAF4 mutation, pathogenic mechanism, autosomal recessive inheritance

## Abstract

Primary ciliary dyskinesia (PCD) is a rare autosomal recessive disorder that affects the structure and function of motile cilia, leading to classic clinical phenotypes, such as situs inversus, chronic sinusitis, bronchiectasis, repeated pneumonia and infertility. In this study, we diagnosed a female patient with PCD who was born in a consanguineous family through classic clinical manifestations, transmission electron microscopy and immunofluorescence staining. A novel *DNAAF4* variant NM_130810: c.1118G>A (p. G373E) was filtered through Whole-exome sequencing. Subsequently, we explored the effect of the mutation on DNAAF4 protein from three aspects: protein expression, stability and interaction with downstream DNAAF2 protein through a series of experiments, such as transfection of plasmids and Co-immunoprecipitation. Finally, we confirmed that the mutation of *DNAAF4* lead to PCD by reducing the stability of DNAAF4 protein, but the expression and function of DNAAF4 protein were not affected.

## Introduction

Primary ciliary dyskinesia (PCD) is a heterogeneous genetic disease that follows the law of autosomal recessive inheritance (Goutaki et al., 2016), although X-linked inheritance has also been reported (Moore et al., 2006). PCD, the first discovered and recognized motile ciliary disease, is classified as a ciliary disease with its incidence rate estimated to be 0.0025%–0.0050% worldwide (Goutaki et al., 2016). PCD is more common among consanguineous families (Lie et al., 2010). Patients with PCD often show some classic clinical phenotypes, such as situs inversus, chronic sinusitis, bronchiectasis, repeated pneumonia and infertility (Knowles et al., 2016).

Cilia exist widely in the kingdom of eukaryotes, and the structure of ciliary axoneme is highly conserved in evolution. According to the structure and function, cilia can be divided into three categories: motile cilia, non-motile cilia (also known as primary cilia) and nodal cilia (Antony et al., 2021). Motile cilia are consisted of a central pair microtubules surrounded by nine doublet microtubules in “9 + 2” pattern. Every single doublet has an inner dynein arm and an outer dynein arm which are protein complex consisted of heavy, intermediate and light chains. The head of the heavy chain has ATPase activity, which can drive ciliary oscillation by hydrolyzing ATP (Omran et al., 2008). The respiratory tract, auris media, eustachian tube, paranasal sinus, oviduct, and vessels of the brain are all covered by epithelial cells with multiple motile cilia, which can drive the flow of liquids and the clearance of foreign objects through precise, regular, coordinated and orderly movement (Mitchison and Valente, 2017). Primary cilia, existing almost all types of cells, are composed of “9 + 0” microtubule ultrastructure without dynein proteins and exert the function of signal transduction (Anvarian et al., 2019). Nodal cilia, composed of “9 + 0” microtubule ultrastructure with dynein proteins, are transiently expressed on the ventral surface of the gastrulation during the period of embryonic development, which can rotate clockwise to make the body fluid around the embryo flow leftward. Embryo senses its position through the flow of body fluid so that organs deflect normally (Hirokawa et al., 2006). The etiology of PCD lies in the structural or functional impairment of the motile cilia. Clinical symptoms of PCD patients are consistent with the distribution of motile cilia in the body.

At present, more than 40 genes have been confirmed to be associated with the pathogenesis of PCD (Bhatt and Hogg, 2020), such as *DNAAF1, DNAAF2, DNAAF3, DNAAF4, DNAH5, DNAH9, and DNALI1.* These genes are involved in the formation of motile cilia. In the case of dynein arms, the IDAs and ODAs need to pre-assembled in the cytoplasm before being transported to the microtubule doublets for assembly by the intraflagellar transport (IFT) (Kozminski et al., 1993; Omran et al., 2008). In this process, dynein axonemal assembly factors play a key role in cytoplasmic pre-assembly steps (Bhatt and Hogg, 2020), including DNAAF1, DNAAF2, DNAAF3, DANNF4, DNAAF5, and LRRC6. Theoretically, any genetic mutation participated in the synthesis, assemble, transport of motile cilia related components could lead to PCD.

In this study, we investigated the clinical and genetic information of a PCD pedigree and further explored the pathogenic mechanism of a novel site mutation in *DNAAF4.*

## Material and methods

### Whole-exome sequencing and variant analysis

DNA extraction kit (QIAamp DNA Blood Midi Kit, Qiagen, Valencia, CA) was used to extract genomic DNA (gDNA) from peripheral blood samples of the proband and her family members. Subsequently, the gDNA of proband was sent to Novogene company (Beijing, China) for Whole-exome Sequencing by Agilent SureSelect Human All Exon V6 kit (Agilent, California, United States) and Illumina platform. We filterd for SNPs and Indels from sequencing data according to the following requirements: 1) Remove mutations with a population frequency higher than 1% in 1000 Genomes, ESP6500, gnomAD_ALL and gnomAD_EAS databases. 2) remain the variants predicted pathogenic by SIFT, MutationTaster, CADD, Polyphen softwares. 3) Classify the variants according to the American College of Medical Genomics (ACMG) guideline of 2015. The variants in patient family members were validated by sanger sequencing. The primer sequences were designed as follows: Forward Sequence ATT​CCT​GCT​CCT​CGC​TCT​GTT​G and Reverse Sequence TGC​TCT​TCG​TGC​CTC​AGC​TTG​T.

We analyzed the evolutionary conservation of DNAAF4 protein by comparing the amino acid sequences in different species found from NCBI database (https://www.ncbi.nlm.nih.gov/).

### Transmission electron microscopy

The bronchial ciliary epithelium sample from the proband was fixed with 2.5% glutaraldehyde in 0.1 M sodium cacodylate buffer and postfixed in 1% osmium tetroxide at 4°C. Following dehydration, the samples were embedded in epoxy resin.

Ultrathin sections stained with 1% uranyl acetate and Reynold’s lead citrate were imaged by HT7700 Hitachi electron microscope (Hitachi, Tokyo, Japan) and NIKON DS-U3 Digital Sight (NIKON, Tokyo, Japan).

### Immunofluorescence

Bronchial ciliary epithelium tissue was obtained by transbronchial lung biopsy. The tissue samples were made into paraffin sections. After being repaired with antigen retrieval solution (PN0012, Pinuofei, China), they were incubated with DNAH9 (PA5-57958, Thermo Fisher, United States), DNAH5 (PA5-45744, Thermo Fisher, United States), DNALI1 (HPA028305, Sigma, United States), anti-acetylated tubulin monoclonal antibody (bsm-33235M, Bioss, China) for 2.5 h and then with secondary antibodies (anti-rabbit IgG, GB22301, Servicebio, China) for 1 h at room temperature. Finally, DAPI (C1002, Beyotime, China) was used to stain nuclei. The pictures were collected by a confocal fluorescence microscope (NIKON, Tokyo, Japan).

### Cell culture and plasmids transfection

HEK293T cells were chosen in all cell experiments in this paper. DMEM (Gibco) with 10% fetal bovine serum (Gibco) and 1% penicillin-streptomycin solution (Servicebio), and environment at 37 °C under humidified 5% CO2 were used to maintain HEK293T cells.

The plasmids including normal and mutant *DNAAF4* plasmids with the HA tag fused to the C-terminus and *DNAAF2* plasmids with the Flag tag fused to the C-terminus were constructed in Wellbio (Changsha, China). Lipofectamine 3000 (Invitrogen) was used for transfection of plasmids according to the instructions. Whole cell extracts were collected for Western blot analysis 48 h after transfection.

### Co-immunoprecipitation

After 48 h of transfection, cell protein was extracted through mix with CoIP lysate buffer for 1 h and centrifugation in 7500 rpm for 30 min at 4°C. Subsequently, 20ul of the supernatant was taken as input, and 20 ul Protein G magic beads (Invitrogen) and 2 ul corresponding antibody were added to the other supernatant overnight at 4°C. Then, magnetic beads were washed with CoIP buffer for 5 times and added with 20 ul 2×SDS solution to boil for Western blot analysis.

### Western blotting

The cell proteins were extracted with lysate buffer, and then the proteins concentrations were determined by using BCA Protein Assay kit (Sigma). The proteins were separated on SDS-PAGE and transferred to PVDF membranes. QuickBlock Blocking Buffer for Western Blot was used to block membranes for 15 min at room temperature. Then, membranes were incubated with primary antibodies, including DNAAF4 (ab229555, Abcam, United Kingdom), Flag (SAB4200071, Sigma) and HA (H6908, sigma), and corresponding secondary antibodies. The target protein bands were visualized by the ECL substrate and analysed by ImageJ software.

### Statistical analysis

The experimental data are all expressed in mean ± standard deviation of at least three independent experiments. All statistical analyses were performed using SPSS (version 22.0, IBM, New York, United States). Statistical significance was defined as *p* < 0.05.

## Results

### Clinical phenotypes

**FIGURE 1:**
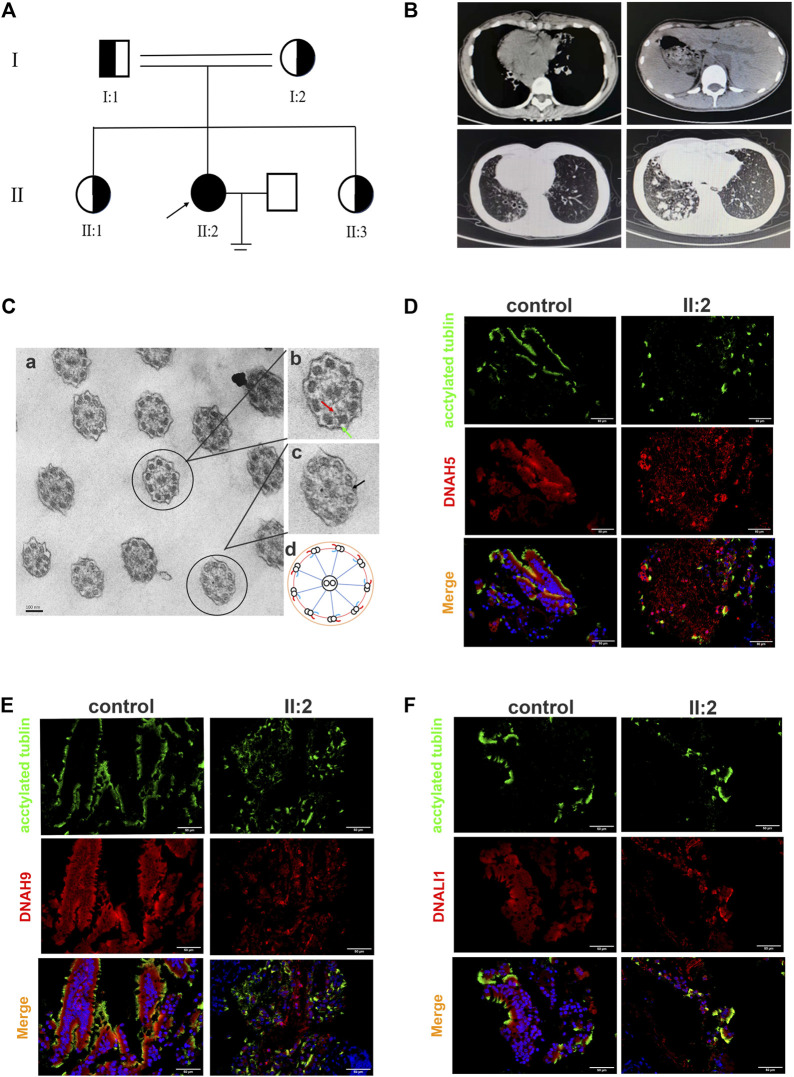
Pedigree of the family with PCD and the clinical features of the proband from this family. **(A)** There is one patient (the proband) in the family with PCD. Circles indicate to females. Squares indicate males. Solid symbols indicate patients. Half solid symbols indicate carriers of the identified mutations. The arrow indicates the proband. **(B)** Chest CT scan of the proband showed complete situs inversus, bronchiectasis and Recurrent bronchitis and pneumonia. **(C)** The transmission electron micrograph (TEM) of bronchial ciliary epithelium from the proband. The red arrow and the green arrow represent IDAs and ODAs, the black arrow indicates absence of IDAs and ODAs **(A–C)**. A schematic diagram of the bronchial ciliary epithelium cross-section**(D)**. **(D–F)** Immunofluorescence of bronchial ciliary epithelium revealed the existence of DNAH5, DNAH9 and DNALI1 of proband. Anti-acetylated tubulin monoclonal antibody was used to mark the ciliary axoneme. DNAH5 and DNAH9 were used to label the outer dynein arm (ODA), DNALI1 was used to label the inner dynein arm (IDA).

The proband (II:2) from a consanguineous family exhibited characteristic clinical manifestations of PCD, including complete situs inversus, chronic sinusitis, bronchiectasis, recurrent bronchitis and pneumonia and infertility (Table 1; Figure 1A). Meanwhile, these symptoms did not occur in her family members. These symptoms are clearly visible on Computed tomography (CT) examination of patient (Figure 1B). Her nasal nitric oxide (nNo) level was 7bpp and pulmonary function test (PFT) revealed severe obstructive ventilatory dysfunction (Table 1). The transmission electron micrograph (TEM) of bronchial ciliary epithelium from the proband showed partial loss of ODAs and IDAs (Figure 1C). Immunofluorescence of bronchial ciliary epithelium showed the existence of composition of ODAs and IDAs (Figures 1D–F). This is consistent with the results of TEM.

**TABLE 1:** Family member phenotypes and genotypes.

Family member	Age/Gender	Clinical findings	Gene	Mutation type	Pulmonary function		
					FEV1%Pre	FEV1/FVC	nNO
I:1	56 years/male	-	*DNAAF4*	Heterozygous	NA	NA	NA
I:2	55 years/female	-	*DNAAF4*	Heterozygous	NA	NA	NA
II:1	34 years/female	-	*DNAAF4*	Heterozygous	NA	NA	NA
II:2	28 years/female	Complete situs inversus, chronic sinusitis, bronchiectasis, Recurrent bronchitis and pneumonia, infertility	*DNAAF4*	Homozygous	46.5%	74.5%	7bpp
II:3	27 years/female	-	*DNAAF4*	Heterozygous	NA	NA	NA

FEV1, forced capacity volume in 1 s; FVC, forced vital capacity; nNO, nasal nitric oxide; NA, not available.

### Whole-exome sequencing and mutation validation

**FIGURE 2:**
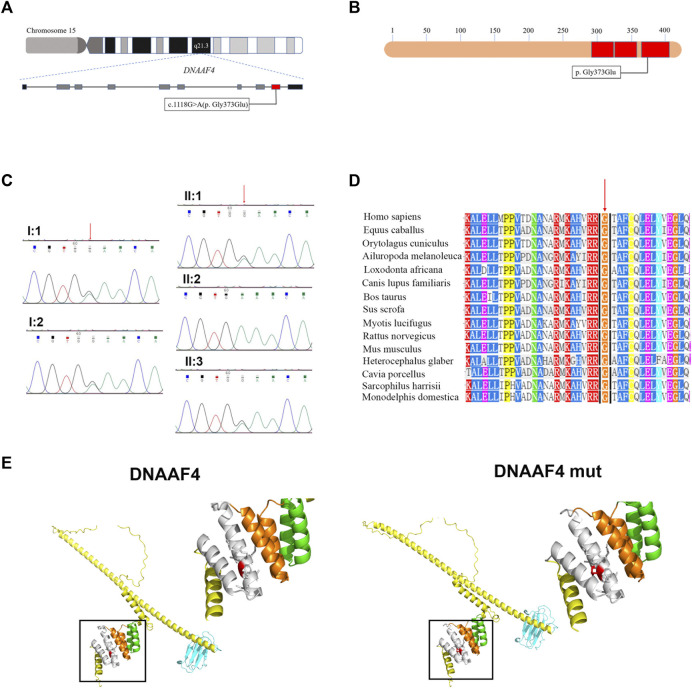
Identification of the *DNAAF4* Mutation. **(A)** Schematic of chromosome 15 and the genomic structure of *DNAAF4*. The red rectangle represents exon9. The boxed Mutation was newly found in this study. **(B)** Schematic showing protein primary structure of DNAAF4; The mutation is located within the TPR domains (red rectangles) at the C terminus of DNAAF4. **(C)** Sanger sequencing chromatogram of proband and her family members. A novel homozygous variant *DNAAF4* **(C)** 1118G>A (p. G373E) was identified in the proband. Her family members were heterozygous at the same position. **(D)** Orthologous protein sequence alignment of DNAAF4 from different species. The red arrow shows that the novel mutation occurred at highly conserved position in these species. **(E)** 3D mock structure of DNAAF4 protein. The white, orange, and green indicate the three TPR domains. The variant p. (G373E) is indicated by red color.

In this study, the blood sample of the proband was performed by whole-exome sequencing (WES). To confirm the accuracy and reliability of the sequencing results, we assessed the quality of the data. The readable genes with coverage depth up to 10× accounted for 99.5% of the reference regions. After filtering SNPs and indels through known databases such as 1000G, dbSNP, YH databases and our inner database, and comparing with currently known PCD causative genes or candidate genes, we identified a homozygous *DNAAF4* variant (NM_130810:exon9:c.1118G>A (p. G373E)) (Figure 2A). This is a completely new mutation that has not been reported before, and the population frequency is extremely low. Subsequently, we performed Sanger sequencing on the mutation site in family members of proband (I:1, I:2, II:1, and II:3), and found that her family members were heterozygous for this variant (Figure 2C), which fits the autosomal recessive model (Figure 1A). The mutation was predicted to cause a missense change in amino acid 373 of the entire 420-amino-acid sequence, which is located in the important functional domain of DNAAF4 protein, the C-terminal tetratricopeptide-repeat (TPR) domain (Figure 2B). Then, we analyzed the conservation of DNAAF4 protein sequence and the pathogenicity of the mutation. Sequence alignment of DNAAF4 protein in human and other orthologs showed that DNAAF4 protein sequence was highly conserved at position of amino acid 373 (Figure 2D). Using alpha Fold to predict the structure of DNAAF4 protein, it was found that the mutation P. (G373E) did not affect the overall spatial structure of the protein, but only affected the local protein conformation, which meant that the mutation may not affect the function of the protein (Figure 2E). We used MutationTaster, SIFT and Polyphen-2 software to predict the pathogenicity of the mutation, demonstrating disease-causing, damaging and probably damaging, and evaluated the mutation as likely pathogenic according to ACMG guideline of 2015, (Table 2).

**TABLE 2:** Pathogenicity prediction of *DNAAF4* Variant.

Gene	NM	Mutation	MutationTaster	SIFT	Polyphen-2	ACMG
*DNAAF4*	NM-130810	c. 1118G>A (p. G373E)	Disease-causing	Damaging	Probably Damaging	PM1+PM2+PP3+PP4

### The mutation of *DNAAF4* reduces the stability of DNAAF4 protein

**FIGURE 3:**
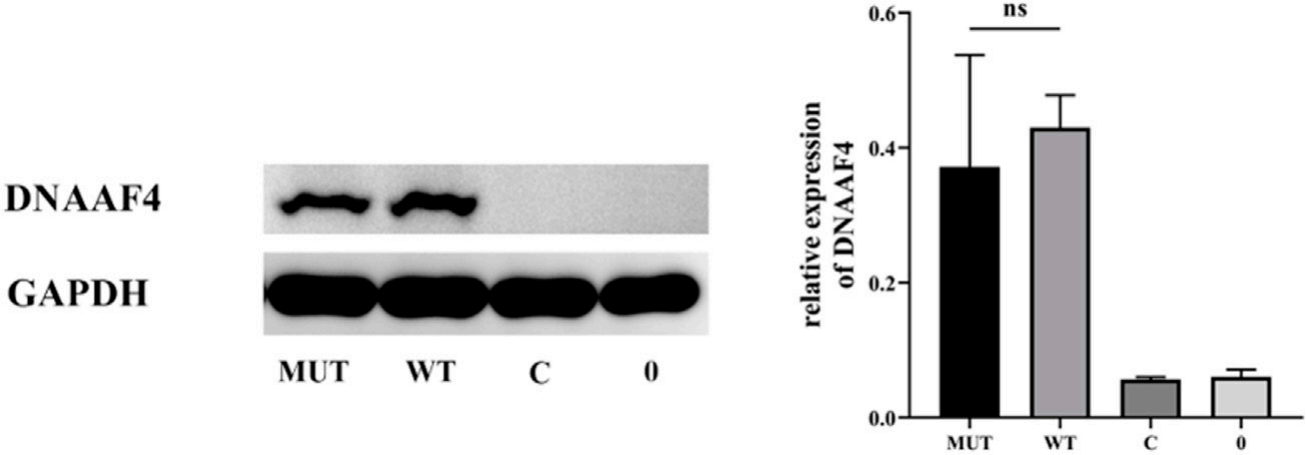
Effect of the Mutation on the expression of the DNAAF4 Protein. Forty-8 hours after transfection of plasmid in HEK293T, Western blot showed the expression of DNAAF4 protein. GAPDH is shown as a reference. MUT, mutated DNAAF4-HA plasmid; WT, normal DNAAF4-HA plasmid; C, blank plasmid; and 0, no plasmid. NS means not significant.

**FIGURE 4:**
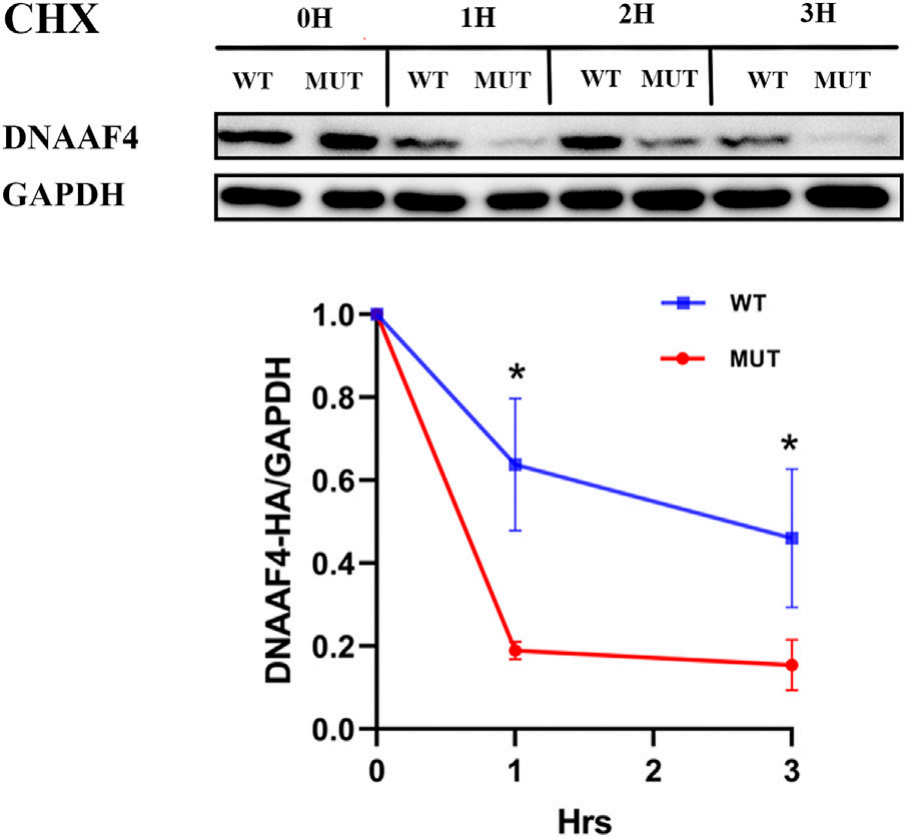
Effect of the Mutation on the Stability of the DNAAF4 Protein. (Top panel) Forty-eight hours after transfection of HEK293T with plasmid, then CHX added, Western blot showed the expression of DNAAF4 protein at 0, 1, 2, and 3 h after addition of CHX. GAPDH is shown as a reference. (Bottom panel) Quantification of the DNAAF4-HA stability relative to GAPDH for this experiment. The densitometric ratio of DNAAF4-HA to GAPDH at the time of addition of CHX is established as 100% and the ratio of the DNAAF4-HA to GAPDH at 1 and 3 h is compared to time 0. *, *p* < 0.05.

**FIGURE 5:**
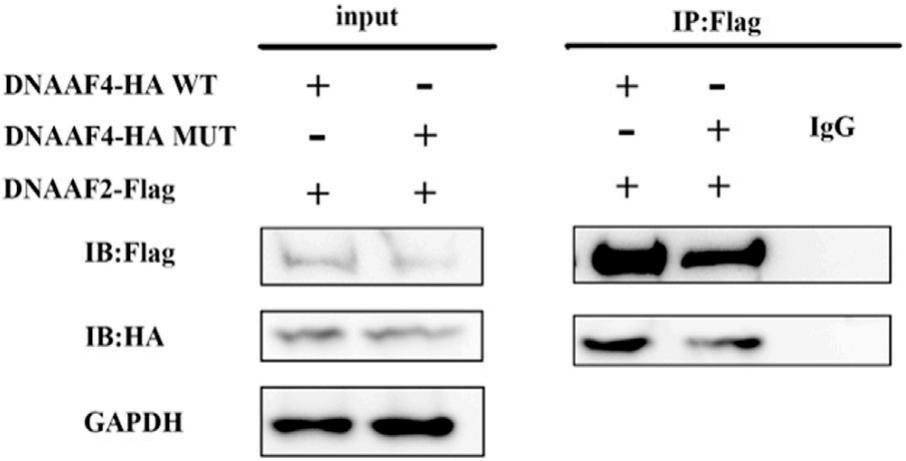
Interaction of the Mutated and Normal DNAAF4 Protein with DNAAF2 protein. DNAAF4 represents a new cytoplasmic axonemal dynein assembly factor, acting together with DNAAF2 at an early step in cytoplasmic ODAs and IDAs assembly. HA-tagged DNAAF4 was co-expressed with Flag-tagged DNAAF2 in HEK293T cells for Forty-eight hours. Extracts were immunoprecipitated with Flag, associated proteins were examined by western blotting using Flag and HA. GAPDH is shown as a reference.

Further, we explored the effect of the mutation on DNAAF4 protein from three aspects: protein expression, stability and interaction with downstream protein. First, We generated *DNAAF4* normal and mutant plasmids with the HA tag fused to the C-terminus of DNAAF4 protein (Supplementary Figure S1), and transfected them into HEK293T cells to ascertain the expression of protein. The levels of DNAAF4 protein extracted from HEK293T cells were detected by western blotting with DNAAF4 antibody. At the same experimental conditions, there was no significant difference in expression between mutant and normal DNAAF4 proteins, and HEK293T cells themselves did not express DNAAF4 protein without transfection of plasmid (Figures 3A,B). Second, To explore the difference in the stability of normal and mutant DNAAF4 proteins, cycloheximide (CHX), a bacterial toxin interfering with protein biosynthesis, was used. We detected the levels of normal and mutant DNAAF4 protein by western blotting with DNAAF4 antibody at 0, 1, 2, and 3 h after CHX added with final concentration of 100 ug/ml, and found that the protein expression of normal and mutant DNAAF4 was approximately equal at 0 h, and the quantity of the mutant protein showed faster reduction than normal protein with time (Figures 4A,B). Third, To study the interaction of DNAAF4 protein and its downstream DNAAF2 protein, we performed the co-immunoprecipitation experiments. We generated *DNAAF2* plasmids with the Flag tag fused to the C-terminus of DNAAF2 protein (Supplementary Figure S1), transferring the *DNAAF4* plasmid and *DNAAF2* plasmid into HEK293T cells for co-expression, and validated that both normal and mutant DNAAF4 proteins can bind to the downstream DNAAF2 protein to exert their physiological functions by western blotting with Flag and HA antibodies (Figure 5). In summary, these experiments confirmed the mutation of *DNAAF4* reduced the stability of DNAAF4 protein.

## Discussion

*DYX1C1* was formerly considered to be a candidate gene for dyslexia (Taipale et al., 2003), but its pathogenesis has not been confirmed. Later, mutations of *DYX1C1* gene connected to cilia structure and motor function were discovered in patients without dyslexia. In 2013, Tarkar (Tarkar et al., 2013) found that mice deleting exons two to four of the *DYX1C1* gene exhibited a PCD phenotype and further confirmed that *DYX1C1* is the disease-causing gene of PCD. *DYX1C1*, now known as *DNAAF4*, is a newly identified dynein axonemal assembly factor that localizes in the cytoplasm of respiratory epithelial cells. It interacts with DNAAF2 to regulate the pre-assembly of IDAs and ODAs. There are only seven pathogenic mutations of *DNAAF4* having been identified and documented in human gene mutation database (HGMD) until now.

*DNAAF4* is located on chromosome 15q21.3 and consists of 10 exons. The DNAAF4 protein has 420 amino acids overall and three TPR domains at its C terminus. The TPR domains play a very important role in the structure and function of DNAAF4 protein, like interacting with molecular chaperones. All of the previously known mutations in DNAAF4 were non-sense mutations, which resulted in premature termination of protein translation before the TPR domains and caused severe clinical phenotypes with defection of ODAs and IDAs (Tarkar et al., 2013). In our study, we analysed and identified a missense mutation in *DNAAF4*, which is located in the TPR domains. WES for the genetic analysis, transmission electron microscopy, and immunofluorescence were used to identify a disease-causing homozygous *DNAAF4* mutation. Then we further studied the effect of the mutation and found that the expression and function of DNAAF4 protein were not affected, but its stability was significantly decreased, which were consistent with the prediction of DNAAF4 protein structure. The instability may lead to insufficient pre-assembly of dynein in the cytoplasm, which also explained the partial absence of ODAs and IDAs in transmission electron microscopy of bronchial ciliary epithelium from the proband.

In PCD patients, different genotypes exhibit different clinical features, ranging from mild to severe phenotypes. Genotype-phenotype relationships have also attracted increasing attention (Brennan et al., 2021). Respiratory symptoms and infertility are the most important clinical phenotypes affecting the quality of life in PCD patients. The severity of respiratory symptoms is related to specific genes. Patients with biallelic mutations of *CCDC39* or *CCDC40*, which encode coiled-coil domain containing proteins to regular the correct spatial structure of IDAs and radial spokes, experienced faster decline in pulmonary function than patients with mutations of *DNAH5* (Davis et al., 2019). Conversely, individuals with biallelic *MNS1* (Ta-Shma et al., 2018)*, DNAH9* (Fassad et al., 2018)*, CFAP53* (Silva et al., 2016) or *RSPH1* (Knowles et al., 2014) mutations have a lower prevalence of neonatal respiratory distress and a later and milder onset of respiratory symptoms. Current studies revealed a link between particular genes and infertility phenotypes. The flagellum of sperm has the “9 + 2” structure similar to the motile cilia. Therefore, the mutations of PCD-related genes will also affect the structure and function of sperm flagellum, leading to infertility. Patients (both male and female) with mutations in the genes *DNAH5* (Aprea et al., 2021), *DNAH11* (Schwabe et al., 2008), *CCDC114* (Onoufriadis et al., 2013) encoding the ODAs and *RSPH4A* (Castleman et al., 2009) encoding the radio-spoke protein are fertile. But absolute infertility is occurred in female with mutations in the genes *DNAAF1* and *LRRC6* encoding dynein axonemal assembly factor (Vanaken et al., 2017). Previous studies and reports have suggested that mutations in *DNAAF4* can lead to infertility (male and female) (Tarkar et al., 2013; Guo T et al., 2022). Therefore, we can infer that the *DNAAF4* mutation caused infertility in the proband. In the clinical phenotype of infertility, patients with different genders exhibit different manifestations for same gene mutations. It’s possible that this is connected to the differential expression of genes in sperms and oviduct epithelial cells. In addition, situs inversus is a indicative phenotype in diagnosis of PCD. Normal visceral placement is driven by the dynein of nodal cilia, so patients with gene mutations encoding non-dynein structural components such as *RSPH1* (Knowles et al., 2014)*, RSPH3* (Jeanson et al., 2015)*,* and *HYDIN* (Olbrich et al., 2012) have not been reported for situs inversus. The mechanism of different pathogenic genes leading to different clinical phenotypes, or even different mutations of the same pathogenic gene leading to different clinical phenotypes (Shoemark et al., 2018) is still unclear, which requires more basic research to explore.

Patients with known *DNAAF4* mutations tend to exhibit severe phenotypes (Tarkar et al., 2013; Guo T et al., 2022), including neonatal respiratory distress, recurrent respiratory symptoms, and infertility. In this study, the proband did not manifest neonatal respiratory distress, but exhibited complete situs inversus, chronic sinusitis, bronchiectasis, recurrent bronchitis and pneumonia, and infertility, with low level of nNO and severe pulmonary ventilation dysfunction. Currently, researches on genotype-phenotype relationships are primarily based on summaries of the reported cases and our study could provide additional references for the genotype-phenotype research of PCD. Therefore, it is very important to further explore and summarize the genotype-phenotype relationships of PCD patients, which can provide personalized medical services for patients.

This study identified and confirmed a novel c.1118G>A (p.G373E) mutation of the *DNAAF4* associated with severe phenotypes, and further explored its pathogenic mechanism. Our findings enrich the spectrum of *DNAAF4* mutations in PCD, which can contribute to the diagnosis and treatment of PCD and advance reproductive genetic counseling.

## Data Availability

The data presented in the study are deposited in the the China National Genebank (CNGB, https://db.cngb.org/cnsa/) repository, accession number CNP0003761.
