# Supplementary material for: Homozygous mutation in DNAAF4 causes primary ciliary dyskinesia in a Chinese family
Source: Front Genet. 2022 Dec 13;13:1087818. doi: 10.3389/fgene.2022.1087818 (PMC9792849; doi:10.3389/fgene.2022.1087818)
Supplement: Supplementary file 1 [file Table1.DOCX]

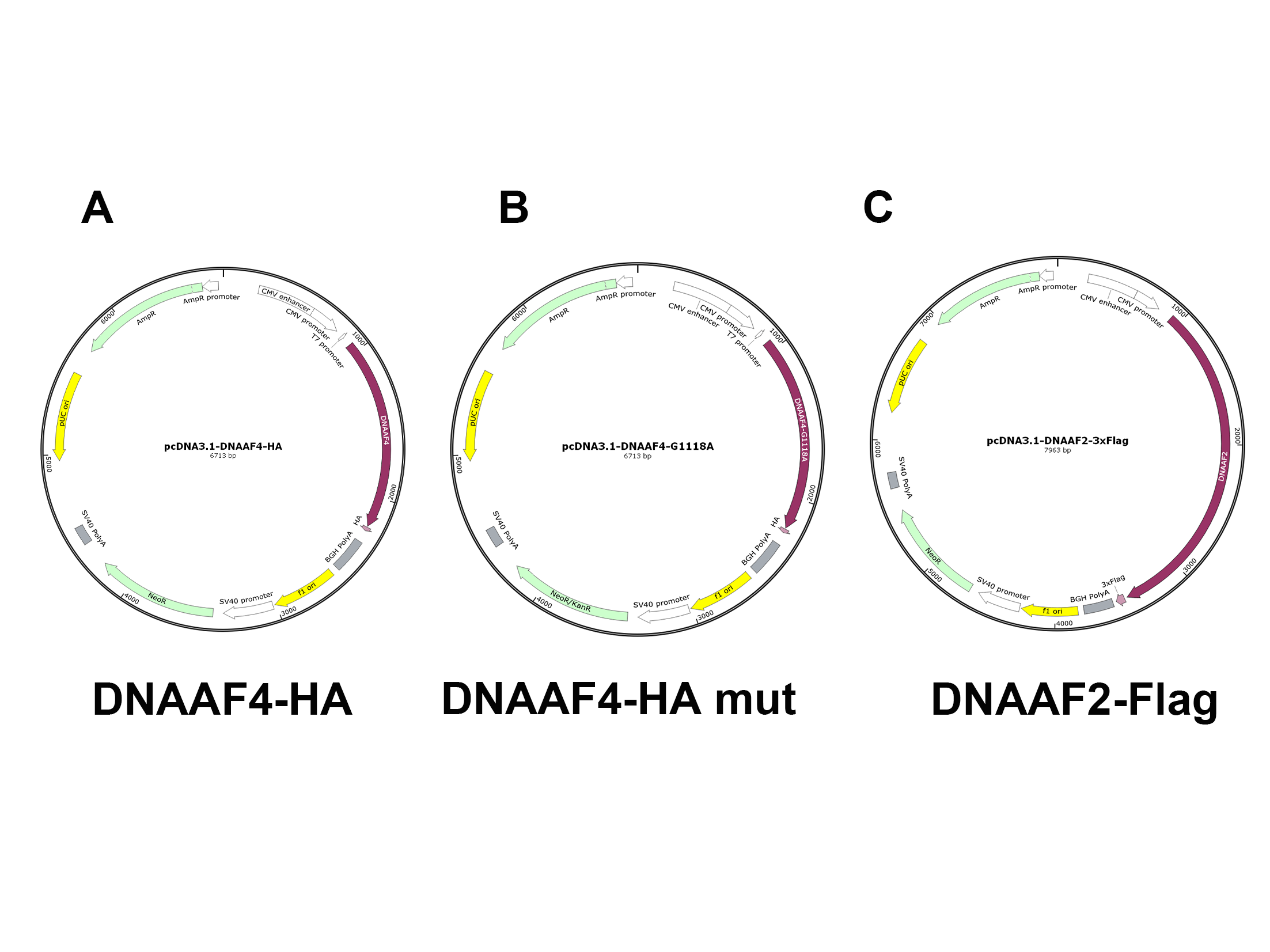


**Figure S1** Plasmid construction patterns. (**A**) Construction pattern of DNAAF4 normal plasmid with the HA tag fused to the C-terminus of DNAAF4 protein. (**B**) Construction pattern of *DNAAF4* mutant plasmid with the HA tag fused to the C-terminus of DNAAF4 protein. (**C**) Construction pattern of DNAAF2 plasmids with the Flag tag fused to the C-terminus of DNAAF2 protein.
